# Analysis of One-Bond Se-Se Nuclear Couplings in Diselenides and 1,2-Diselenoles on the Basis of Molecular Orbital Theory: Torsional Angular Dependence, Electron Density Influence, and Origin in ^1^ *J*(Se, Se)

**DOI:** 10.1155/2009/381925

**Published:** 2009-08-06

**Authors:** Akito Tanioku, Satoko Hayashi, Waro Nakanishi

**Affiliations:** Department of Material Science and Chemistry, Faculty of Systems Engineering, Wakayama University, 930 Sakaedani, Wakayama 640-8510, Japan

## Abstract

Nuclear couplings for the Se-Se bonds, ^1^ *J*(Se, Se), are analyzed on the basis of the molecular orbital (MO) theory. The values are calculated by employing the triple *ζ* basis sets of the Slater type at the DFT level. ^1^ *J*(Se, Se) are calculated modeled by MeSeSeMe (**1a**), which shows the typical torsional angular dependence on *ϕ*(C_Me_SeSeC_Me_). The dependence explains well the observed ^1^ *J*_obsd_ (Se, Se) of small values (≤ 64 Hz) for RSeSeR′ (**1**) (simple derivatives of **1a**) and large values (330–380 Hz) observed for 4-substituted naphto[1,8-*c*, *d*]-1,2-diselenoles (**2**) which correspond to *symperiplanar* diselenides. ^1^ *J* (Se, Se: **2**) becomes larger as the electron density on Se increases. The paramagnetic spin-orbit terms contribute predominantly. The contributions are evaluated separately from each MO (*ψ*_*i*_) and each *ψ*_*i*_ → *ψ*_*a*_ transition, where *ψ*_*i*_ and *ψ*_*a*_ are occupied and unoccupied MO's, respectively. The separate evaluation enables us to recognize and visualize the origin and the mechanism of the couplings.

## 1. Introduction

Indirect nuclear spin-spin coupling constants (*J*) provide highly important information around coupled nuclei, containing strongly bonded and weakly interacting states, since the values depend on the electron distribution between the nuclei [1–10]. One–bond (^1^ *J*), two-bond (*geminal*) (^2^ *J*), three-bond (*vicinal*) (^3^ *J*), and even longer coupling constants (^*n*^ *J* (*n* ≥ 4)) are observed between selenium atoms, which will give important information around the coupled nuclei. The mechanism for ^1^ *J* must be of the through-bond type; however, that for ^*n*^ *J* (*n* ≥ 2) would contain through-space interactions, especially for ^*n*^ *J* (*n* ≥ 4). Quantum chemical (QC) calculations are necessary for the analysis and the interpretation of the *J* values with physical meanings. Important properties of molecules will be clarified by elucidating the mechanism of spin-spin couplings on the basis of the molecular orbital (MO) theory.

**Figure 1:**
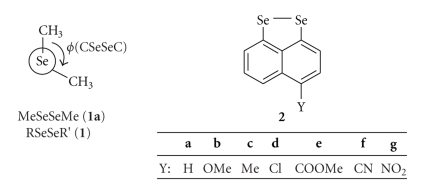


Various ^1^ *J*_obsd_ (Se, Se) values are reported for alkyl and/or aryl derivatives of dimethyl diselenide (**1a**) (RSeSeR′: **1**). They are usually small (^1^ *J*_obsd_ (Se, Se: **1**) ≤ 64 Hz; see Table 1). We examined ^1^ *J*(Se, Se) of naphto[1,8-*c,d*]-1,2-diselenole (4-Y-1,8-Se_2_C_10_H_5_ (**2**): Y = H (**a**) [11–15], OMe (**b**), Me (**c**), Cl (**d**), COOMe (**e**), CN (**f**), and NO_2_ (**g**)), which correspond to the *symperiplanar* diselenides (Figure 1). The ^1^ *J*(Se, Se) values are measured for **2c**, **2d**, and **2g**, and large ^1^ *J*_obsd_ (Se, Se) values of 330–380 Hz are detected. Table 1 summarizes the ^1^ *J*_obsd_ (Se, Se) values.

Why are ^1^ *J*_obsd_ (Se, Se: **2**) much larger than ^1^ *J*_obsd_ (Se, Se: **1**)? How do ^1^ *J*_obsd_ (Se, Se: **2**) depend on the substituent Y in **2**? ^1^ *J*(Se, Se) are analyzed on the basis of the MO theory, as the first step to investigate the nature of the bonded and nonbonded interactions between selenium atoms through ^*n*^ *J*(Se, Se) [18]. ^1^ *J*(Se, Se) are calculated for **1a** and **2a**–**g**.

According to the nonrelativistic theory, there are several mechanisms contributing to the spin-spin coupling constants. As expressed in (1), the total value (^*n*^ *J*_TL_) is composed of the contributions from the diamagnetic spin-orbit (DSO) term (^*n*^ *J*_DSO_), the paramagnetic spin-orbit (PSO) term (^*n*^ *J*_PSO_), the spin-dipolar (SD) term (^*n*^ *J*_SD_), and the Fermi contact (FC) term (^*n*^ *J*_FC_),

(1) JTLn=JDSOn+JPSOn+JSDn+JFCn.

**Scheme 1:**
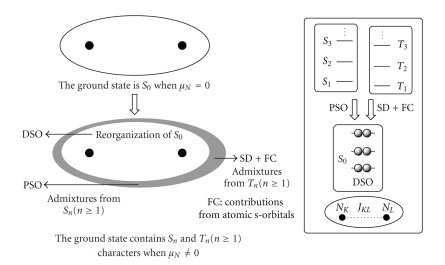
How do the indirect nuclear spin-spin couplings originate? Mechanisms for ^*n*^ *J*_DSO_, ^*n*^ *J*_PSO_, ^*n*^ *J*_SD_, and ^*n*^ *J*_FC_ terms, contributing to ^*n*^ *J*_TL_.

Scheme 1  summarizes the mechanism of the indirect nuclear spin-spin couplings. The origin of the terms, ^*n*^ *J*_DSO_, ^*n*^ *J*_PSO_, ^*n*^ *J*_SD_, and ^*n*^ *J*_FC_, is also illustrated, contributing to ^*n*^ *J*_TL_. The ground state of a molecule (***M***) is the singlet state (*S*_0_) if the nuclei (*N*) in ***M*** have no magnetic moments. However, the ground state cannot be the pure *S*_0_ if *N* possesses magnetic moments, *μ*_*N*_. The ground state perturbed by *μ*_*N*_ is expressed as follows: DSO arise by the reorganization of *S*_0_; therefore, they are usually very small. PSO appears by the mixing of upper singlet states (*S*_1_, *S*_2_, *S*_3_,…). FC and SD originate if admixtures occur from upper triplet states (*T*_1_, *T*_2_, *T*_3_,…), where only s-type atomic orbitals contribute to FC.

Calculated ^1^ *J*_TL_ values are evaluated separately by the four components as shown in (1). The ^1^ *J*(Se, Se) values are evaluated using the Slater-type atomic orbitals, which are equipped in the ADF 2008 program [19–23]. Evaluations of the values are performed employing the ADF program, after structural optimizations with the Gaussian 03 program [24]. Contributions from each *ψ*_*i*_ and each *ψ*_*i*_ → *ψ*_*a*_ transition are evaluated separately, where *ψ*_*i*_ and *ψ*_*a*_ denote occupied and unoccupied MOs, respectively. The treatment enables us to recognize and visualize clearly the origin of the indirect nuclear spin-spin couplings.

## 2. Experimental

### 2.1. Materials and Measurements

Manipulations were performed under an argon atmosphere with standard vacuum-line techniques. Glassware was dried at 130°C overnight. Solvents and reagents were purified by standard procedures as necessary. Melting points were measured with a Yanaco-MP apparatus of uncorrected. Flash column chromatography was performed on silica gel (Fuji Silysia PSQ-100B), acidic and basic alumina (E. Merck). 

NMR spectra were recorded at 297 K in CDCl_3_ and DMSO-*d*_6_ solutions. ^1^H, ^13^C, and ^77^Se NMR spectra were measured at 300, 75.5, and 76.2 MHz, respectively. Chemical shifts are given in ppm relative to those of TMS for ^1^H and ^13^C NMR spectra and relative to reference compound Me_2_Se for ^77^Se NMR spectra.

### 2.2. Preparation of 4-methylnaphtho[1,8-c,d]-1,2-diselenole (2b)

According to a method similar to that previously reported for **2a** [11–17] from 1,8-dichloro-4-methylnaphthalene, **2b** was obtained as purple needles in 68% yield, m.p. 127.0–128.0°C. ^1^H NMR (CDCl_3_, 300 MHz, TMS): *δ* 2.50 (s, 3H), 7.09 (dd, 1H, *J* = 0.9 and 7.6 Hz), 7.25 (d, 1H, *J* = 7.3 Hz), 7.36 (dd, 1H, *J* = 0.6 and 6.9 Hz), 7.55 (dd, 1H, *J* = 0.7 and 8.4 Hz); ^13^C NMR (CDCl_3_, 75.5 MHz, TMS): *δ* 18.6, 120.4, 120.7, 121.0, 127.4, 128.2, 130.4, 137.0, 137.3, 138.0, 141.1; ^77^Se NMR (CDCl_3_, 76.2 MHz, Me_2_Se): *δ* 411.8, 420.6. Anal. Calc. for C_11_H_8_Se_2_: C, 44.32; H, 2.70%; found: C, 44.21; H, 2.63%.

### 2.3. Preparation of 4-chloronaphtho[1,8-c,d]-1,2-diselenole (2c)

According to a method similar to that previously reported for **2a** [11–17] from 1,4,8-trichloronaphthalene, **2c** was obtained as brown needles in 58% yield, m.p. 155.0–156.0°C. ^1^H NMR (CDCl_3_, 300 MHz, TMS): *δ* 7.24 (d, 1H, *J* = 8.1 Hz), 7.30 (d, 1H, *J* = 7.9 Hz), 7.34 (t, 1H, *J* = 7.7 Hz), 7.39 (dd, 1H, *J* = 1.2 and 7.4 Hz), 7.81 (dd, 1H, *J* = 1.3 and 7.9 Hz); ^13^C NMR (CDCl_3_, 75.5 MHz, TMS): *δ* 120.5, 120.6, 121.9, 127.3, 127.4, 128.6, 135.0, 138.5, 140.0, 141.2; ^77^Se NMR (CDCl_3_, 76.2 MHz, Me_2_Se): *δ* 422.6, 444.6. Anal. Calc. for C_10_H_5_Se_2_Cl: C, 37.71; H, 1.58%; found: C, 37.83; H, 1.60%.

### 2.4. Preparation of 4-nitronaphtho[1,8-c,d]-1,2-diselenole (2d)

According to a method similar to that previously reported for **2a** [11–17] from 1,8-dibromo-4-nitronaphthalene, **2d** was obtained as dark purple needles in 28% yield, m.p. 196.0–197.0°C. ^1^H NMR (CDCl_3_, 300 MHz, TMS): *δ* 7.40 (d, 1H, *J* = 8.3 Hz), 7.52 (dd, 1H, *J* = 4.1 and 7.6 Hz), 7.53 (s, 1H), 8.18 (d, 1H, *J* = 8.3 Hz), 8.51 (dd, 1H, *J* = 2.7 and 4.1 Hz); ^1^H NMR (DMSO-d_6_, 300 MHz, TMS): *δ* 7.57 (dd, 1H, *J* = 7.5 and 8.5 Hz), 7.77 (d, 1H, *J* = 8.5 Hz), 7.84 (dd, 1H, *J* = 0.7 and 7.5 Hz), 8.20 (d, 1H, *J* = 8.5 Hz), 8.29 (dd, 1H, *J* = 0.7 and 8.5 Hz); ^13^C NMR (DMSO-d_6_, 75.5 MHz, TMS): *δ* 118.2, 120.0, 123.4, 127.1, 129.4, 131.1, 139.0, 140.8, 144.2, 155.5; ^77^Se NMR (CDCl_3_, 76.2 MHz, Me_2_Se): *δ* 448.8, 474.4. Anal. Calc. for C_10_H_5_Se_2_NO_2_: C, 36.50; H, 1.53; N, 4.26%; found: C, 36.41; H, 1.40; N, 4.19%.

### 2.5. Measurements of ^1^ *J*(Se, Se)

During the measurement of ^77^Se NMR spectra for **2g** (Y = NO_2_) in chloroform-*d* solutions (0.050 M) at 297 K, a typical AB quartet pattern of the spectra was observed. After careful analysis of the spectrum for **2g**, ^1^ *J*(Se, Se) of 330.8 Hz was obtained. The ^1^ *J*(Se, Se) values are obtained similarly by the careful analysis of the spectra for **2c** and **2d**.

### 2.6. Calculation Method

Structures of **1a** are optimized employing the 6-311++G(3df,2pd) basis sets of the Gaussian 03 program [24–28] at the DFT (B3LYP) level [29–32]. The torsional angle C_Me_SeSeC_Me_ (*ϕ*) is 88.38° in the full-optimized structure of **1a**. Calculations that are further performed on **1a**: **1a** are fully optimized except for *ϕ*, which are fixed by every 15° or 30°. Optimizations are also performed on **2a–g** using the 6-311+G(3df) basis sets [25–28] for Se and the 6-311+G(3d,2p) basis sets for other nuclei at the DFT (B3LYP) level [29–32]. The *C*_2*v*_ symmetry is assumed for **2a**, *C*_*s*_ for **2b**–**d** and **2f**, and the *C*_1_ symmetry for **2e** and **2g**. 

The *J*(Se, Se) values are calculated with the triple *ξ* basis sets of the Slater type with two sets of polarization functions (2 × 1s, 2 × 2s, 2 × 2p, 2 × 3s, 2 × 3p, 3 × 3d, 3 × 4s, 3 × 4p, 1 × 4d, and 1 × 4f for Se) at the DFT (BLYP) level of the ADF 2008 program [19–23], applying on the optimized structures with the Gaussian 03 program [24]. Calculations are performed at the nonrelativistic level. The scalar ZORA relativistic formulation [33–35] is also applied to **2a**, for convenience of comparison. The ^*n*^ *J*_TL_ values are evaluated separately by ^*n*^ *J*_DSO_, ^*n*^ *J*_PSO_, ^*n*^ *J*_SD_, and ^*n*^ *J*_FC_, as shown in (1). Mechanisms of the nuclear couplings are revealed by decomposing the contributions to each *ψ*_*i*_ and each *ψ*_*i*_ → *ψ*_*a*_ transition [36, 37].

## 3. Results and Discussion

### 3.1. Observed ^1^ *J*_obsd_ (Se, Se)

Table 1 collects ^1^ *J*_obsd_ (Se, Se), necessary for discussion. The magnitudes of the ^1^ *J*_obsd_ (Se, Se) values are usually small (< 64 Hz) for the simple derivatives of MeSeSeMe (**1a**) (RSeSeR′: **1**) [9, 16, 17]. On the other hand, large ^1^ *J*_obsd_ (Se, Se) are recorded for **2** (4-Y-1,8-Se_2_C_10_H_5_), which correspond to *symperiplanar* diselenides, although not detected in **2a** (Y = H) [11–15]. The values are 379.4 Hz for **2b** (Y = Me), 375.9 Hz for **2c** (Y = Cl), and 330.8 Hz for **2d** (Y = NO_2_). ^1^ *J*(Se, Se: **2**) becomes smaller as the electron accepting ability of Y increases.

### 3.2. Mechanism of ^1^ *J*(Se, Se) in 1a

**Figure 2:**
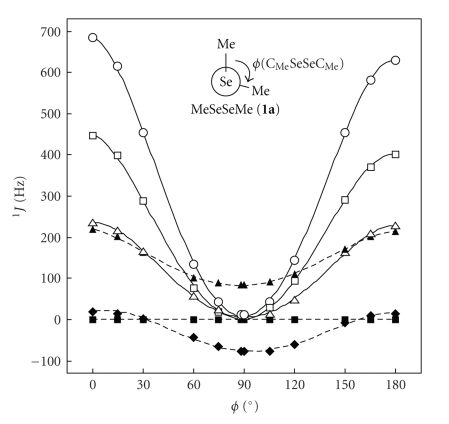
Plots of ^1^ *J*_DSO_ (■),^1^ *J*_PSO_ (□),^1^ *J*_SD_ (▲),^1^ *J*_FC_ (♦),^1^ *J*_SD+FC_ (△), and ^1^ *J*_TL_ (○) versus *ϕ*(CSeSeC) in ^1^ *J*(Se, Se: **1a**).

Table 2 shows the calculated ^1^ *J*_TL_ and the components, ^1^ *J*_DSO_, ^1^ *J*_PSO_, ^1^ *J*_SD_, and ^1^ *J*_FC_, in ^1^ *J*(Se, Se: **1a**). ^1^ *J*_TL_ (Se, Se: **1a**) is predicted to be less than 44 Hz for *ϕ* = 90° ± 15°. Therefore, ^1^ *J*_obsd_ (Se, Se: **1**) is explained substantially and modeled by **1a** with *ϕ* ≈ 90°, although R and R′ in **1** must also affect on the values. ^1^ *J*(Se, Se: **1a**) is predicted to be very large at *ϕ* = 0° (684 Hz) and 180° (628 Hz). Consequently, ^1^ *J*_obsd_ (Se, Se: **2**) of 331–379 Hz are essentially explained by ^1^ *J*(Se, Se: **1a**) with *ϕ* = 0°. Figure 2 draws the plots of ^1^ *J*_DSO_, ^1^ *J*_PSO_, ^1^ *J*_SD_, ^1^ *J*_FC_, ^1^ *J*_SD+FC_, and ^1^ *J*_TL_ versus *ϕ* in **1a**. It is well demonstrated that ^1^ *J*_TL_ changes depending on *ϕ*, similarly to the case of ^3^ *J*(H, H), three-bond (*vicinal*) couplings in ^1^H NMR spectra [1, 2]. ^1^ *J*_DSO_ are negligible (< 0.03 Hz).

How do ^1^ *J*_PSO_ (Se, Se: **1a**) and ^1^ *J*_SD+FC_ (Se, Se: **1a**) [=^1^ *J*_SD_ (Se, Se: **1a**) + ^1^ *J*_FC_ (Se, Se: **1a**)] contribute to ^1^ *J*_TL_ (Se, Se: **1a**)? ^1^ *J*_PSO_ (Se, Se: **1a**) and ^1^ *J*_SD+FC_ (Se, Se: **1a**) are plotted versus ^1^ *J*_TL_ (Se, Se: **1a**), although not shown. The correlations are given in (2) and (3), respectively. The results exhibit that ^1^ *J*_PSO_ (Se, Se: **1a**) and ^1^ *J*_SD+FC_ (Se, Se: **1a**) contribute 65% and 35% to ^1^ *J*_TL_ (Se, Se: **1a**), respectively, irrespective of the *ϕ*(CSeSeC) values:

(2) J1PSO(Se,Se:  1a)=0.651×J1TL(Se,Se:  1a)−4.1  (r2=0.999),

(3) J1SD+FC(Se,Se:  1a)=0.349×J1TL(Se,Se:  1a)+4.2  (r2=0.998).

Why does ^1^ *J*(Se, Se: **1a**) show the torsional angular dependence? What orbitals and transitions contribute to the dependence? ^1^ *J*_PSO_ (Se, Se: **1a**) is analyzed next.

#### 3.2.1. Analysis of ^1^ *J*_PSO_ (Se, Se) in 1*a*

**Figure 3:**
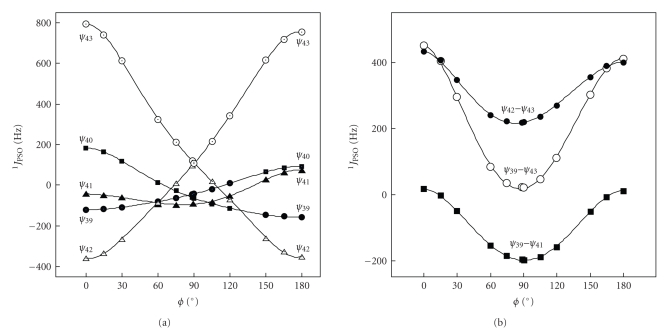
Origin of the torsional angular dependence in ^1^ *J*_PSO_ (Se, Se: **1a**): (a) contributions from each of *ψ*_39_, *ψ*_40_, *ψ*_41_, *ψ*_42_, and *ψ*_43_ and (b) those from *ψ*_39_–*ψ*_41_,*ψ*_42_–*ψ*_43_, and *ψ*_39_–*ψ*_43_.

The mechanism of ^1^ *J*_PSO_ (Se, Se: **1a**) is discussed by analyzing the contributions separately from each *ψ*_*i*_ and each *ψ*_*i*_ → *ψ*_*a*_ transition. Table 3 lists the *ϕ* dependence of ^1^ *J*_PSO_ (Se, Se: **1a**) contributed from *ψ*_1_–*ψ*_43_, *ψ*_1_–*ψ*_38_, *ψ*_39_–*ψ*_43_, *ψ*_39_, *ψ*_40_, *ψ*_41_, *ψ*_42_, and *ψ*_43_. The contribution from *ψ*_39_–*ψ*_43_ to ^1^ *J*_PSO_ (Se, Se: **1a**) is large, whereas that from *ψ*_1_–*ψ*_38_ is small, although not shown. The plot of the contributions from *ψ*_39_–*ψ*_43_ (*y*) versus those from *ψ*_1_–*ψ*_43_ (*x*) provides an excellent correlation (*y* = 0.976*x* + 37.3 : *r* ^2^ = 0.9999). Figure 3(a) shows those from *ψ*_39_, *ψ*_40_, *ψ*_41_, *ψ*_42_, and *ψ*_43_ and Figure 3(b) exhibits those from *ψ*_39_–*ψ*_41_, *ψ*_42_–*ψ*_43_, and *ψ*_39_–*ψ*_43_. Contributions from *ψ*_42_ and *ψ*_43_ exchange with each other at *ϕ* ≈ 90°. Those of *ψ*_39_ and *ψ*_40_ do at *ϕ* ≈ 135° (Figure 3(a)). The contributions from *ψ*_42_-*ψ*_43_ and *ψ*_39_–*ψ*_41_ almost cancel out at *ϕ* ≈ 90° (Figure 3(b)).

Magnitudes of the contributions from *ψ*_42_ and *ψ*_43_ to ^1^ *J*_PSO_ (Se, Se: **1a**) are very large at 0° and 180° (Table 3), although those from *ψ*_42_ and *ψ*_43_ are negative and positive directions, respectively. The values amount to −353 to −360 Hz and 753–793 Hz, respectively. The contributions from *ψ*_42_–*ψ*_43_ are 433, 218, and 400 Hz at 0°, 90°, and 180°, respectively, and those from *ψ*_39_–*ψ*_41_ are 17, −198, and 10 Hz at 0°, 90°, and 180°, respectively. Therefore, the mechanism of ^1^ *J*_PSO_ (Se, Se: **1a**) will be clarified by analyzing the contributions from *ψ*_42_ and *ψ*_43_ at 0° and 180°. The mechanism would be complex at 90°, since the small magnitude is the results of the total contributions from *ψ*_39_–*ψ*_43_.

**Figure 4:**
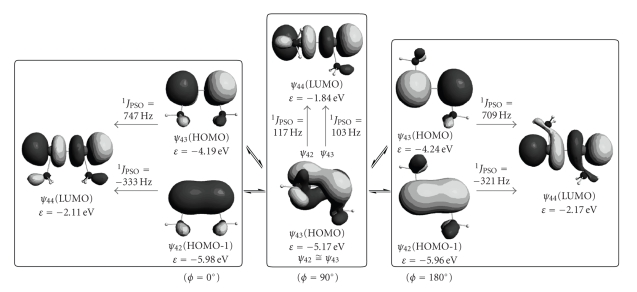
Contributions to ^1^ *J*_PSO_ (Se, Se: **1a**) from the *ψ*_42_ → *ψ*_44_ and *ψ*_43_ → *ψ*_44_ transitions at *ϕ* = 0, 90, and 180°. The interconversion between *ψ*_42_ and *ψ*_43_ at *ϕ* ≈ 90° is also depicted.

Figure 4 shows the *ψ*_42_ → *ψ*_44_ and *ψ*_43_ → *ψ*_44_ transitions at both *ϕ* = 0° and 180° which are shown in Table 3. Characters of *ψ*_42_(HOMO-1), *ψ*_43_(HOMO), and *ψ*_44_(LUMO) are *π*(Se–Se), π*(Se–Se), and σ*(Se–Se), respectively, at *ϕ* = 0° and 180°. *ψ*_42_(HOMO-1) is essentially the same as *ψ*_43_(HOMO) at *ϕ* = 90°. *ψ*_42_ and *ψ*_43_ at *ϕ* = 90° are also drawn in Figure 4, to show how *ψ*_42_ and *ψ*_43_ interconvert with each other. Contrary to the case of *ϕ* ≈ 0 and 180°, all of *ψ*_39_–*ψ*_43_ contribute to ^1^ *J*_PSO_ (Se, Se: **1a**) at *ϕ* ≈ 90°. Contributions from the *ψ*_42_ → *ψ*_44_ and *ψ*_43_ → *ψ*_44_ transitions to ^1^ *J*_PSO_ (Se, Se: **1a**) at 90° are almost cancelled by those from the *ψ*_39_ → *ψ*_44_, *ψ*_40_ → *ψ*_44_, and *ψ*_41_ → *ψ*_44_ transitions. In addition, both ^1^ *J*_SD_ (Se, Se: **1a**) and ^1^ *J*_FC_ (Se, Se: **1a**) substantially contribute at *ϕ* ≈ 90°. Consequently, it is difficult to specify a few orbitals, together with the transitions, which control ^1^ *J*(Se, Se: **1a**) at *ϕ* ≈ 90°. The character of *ψ*_44_ [LUMO: σ*(Se–Se)] does not change so much depending on *ϕ*. Therefore, the behavior of *ψ*_39_–*ψ*_43_ must be mainly responsible for the *ϕ* dependence in ^1^ *J*(Se, Se: **1a**) (see Figures 3  and 4). The MO description in Figure 4 visualizes the origin of ^1^ *J*_PSO_ (Se, Se: **1a**) and helps us to understand the mechanism, especially at *ϕ* = 0° and 180°.

After elucidation of the mechanism for ^1^ *J*_PSO_ (Se, Se: **1a**), next extension is to clarify ^1^ *J*(Se, Se: **2**) on the basis of the MO theory.

#### 3.2.2. Evaluation of ^1^ *J*(Se, Se) for 2

Table 4 collects the calculated ^1^ *J*_TL_ (Se, Se: **2**) values, together with *J*_PSO_(Se, Se: **2**), ^1^ *J*_SD_ (Se, Se: **2**), ^1^ *J*_FC_ (Se, Se: **2**), and ^1^ *J*_SD+FC_ (Se, Se: **2**). Table 4 also contains the nuclear changes calculated with the natural bond orbital analysis (NBO) method (*Qn*(Se)) [38–40] for **2** having Y of H (**a**), OMe (**b**), Me (**c**), Cl (**d**), COOMe (**e**), CN (**f**), and NO_2_ (**g**). The Y dependence of ^1^ *J*_obsd_ (Se, Se: **2**) is well reproduced by the calculations. ^1^ *J*_TL_ (Se, Se: **2**) are predicted to be larger than the observed values by about 100 Hz. The DFT method overestimates the reciprocal energy differences (*ε*_*a*_ − *ε*_*i*_)^−1^, which would partly be responsible for the larger evaluation. The ^1^ *J*(Se, Se) values are calculated at both nonrelativistic and scalar ZORA relativistic levels for **2a**. The former is smaller than the latter. The value calculated at the nonrelativistic level seems to be closer to the observed value than that obtained with the scalar ZORA relativistic formulation in our calculation system. Therefore, it would be reasonable to discuss the ^*n*^ *J*(Se, Se) value calculated at the nonrelativistic level in this case.

**Figure 5:**
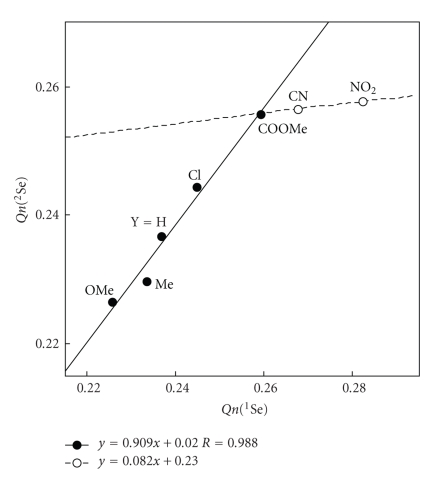
Plot of *Qn*(^2^Se) versus *Qn*(^1^Se) in **2**.

Before discussion of ^1^ *J*(Se, Se: **2**), it would be instructive to clarify the behavior of *Qn*(Se: **2**), which changes depending on Y. Figure 5 shows the plot of *Qn*(^2^Se: **2**) versus *Qn*(^1^Se: **2**). The correlations of the linear type (*y* = *ax* + *b* with *r* (correlation coefficient)) are given in the figure. The results show that *Qn*(^2^Se: **2**) grows larger as the accepting ability of Y increases for Y = H, OMe, Me, Cl, and COOMe then it becomes almost constant for Y = CN and NO_2_ while *Qn*(^1^Se: **2**) grows larger as the accepting ability of Y increases for all Y in Table 4. *Qn*(^2^Se: **2**) seems saturated for Y of very strong acceptors such as CN and NO_2_ while *Qn*(^1^Se: **2**) will not for all Y.

**Figure 6:**
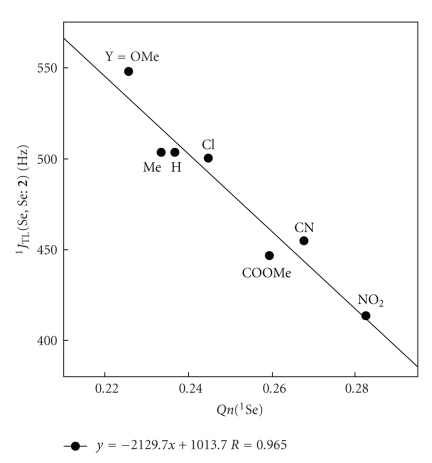
Plot of ^1^ *J*(Se, Se: **2**) versus *Qn*(^1^Se) in **2**.

How do ^1^ *J*_TL_ (Se, Se: **2**) being controlled? ^1^ *J*_TL_ (Se, Se: **2**) are plotted versus *Qn*(^1^Se), * Qn*(^2^Se), and *Qn*(^1^Se)+*Qn*(^2^Se). Figure 6 shows the plot of ^1^ *J*_TL_ (Se, Se: **2**) versus *Qn*(^1^Se), which gives best correlation among the three. The correlation is given in the figure. ^1^ *J*_TL_ (Se, Se: **2**) are confirmed to be controlled by *Qn*(^1^Se). One might imagine that ^1^ *J*_TL_ (Se, Se: **2**) should be controlled by *Qn*(^1^Se)+*Qn*(^2^Se). The saturation in *Qn*(^2^Se) shown in Figure 5 would perturb to give good correlations for ^1^ *J*_TL_ (Se, Se: **2**) versus *Qn*(^1^Se) + *Qn*(^2^Se). It is demonstrated that ^1^ *J*_TL_ (Se, Se: **2**) becomes smaller when *Qn*(Se) increases, experimentally and theoretically.

After clarification of the Y dependence in ^1^ *J*_TL_ (Se, Se: **2**), next extension is to elucidate the mechanism for ^1^ *J*(Se, Se: **2**) on the basis of the MO theory.

### 3.3. Mechanism of ^1^ *J*(Se, Se) in 2*a*

How do ^1^ *J*_PSO_ (Se, Se: **2**) and ^1^ *J*_SD+FC_ (Se, Se: **2**) contribute to ^1^ *J*_TL_ (Se, Se: **2**) in the change of Y? ^1^ *J*_PSO_ (Se, Se: **2**) and ^1^ *J*_SD+FC_ (Se, Se: **2**) are plotted versus ^1^ *J*_TL_ (Se, Se: **2**) for various Y in Table 4. The results for ^1^ *J*_PSO_ (Se, Se: **2**) and ^1^ *J*_SD+FC_ (Se, Se: **2**) are given in (4) and (5), respectively. The correlations are very good, which shows that ^1^ *J*_PSO_ (Se, Se: **2**) contributes predominantly to ^1^ *J*_TL_ (Se, Se: **2**) (70%), irrespective of Y:

(4) JPSO1(Se,Se:  2)=0.704×J1TL(Se,Se:  2)+8.3  (r2=0.999),

(5) JSD+FC1(Se,Se:  2)=0.295×J1TL(Se,Se:  2)−8.0  (r2=0.994)

**Figure 7:**
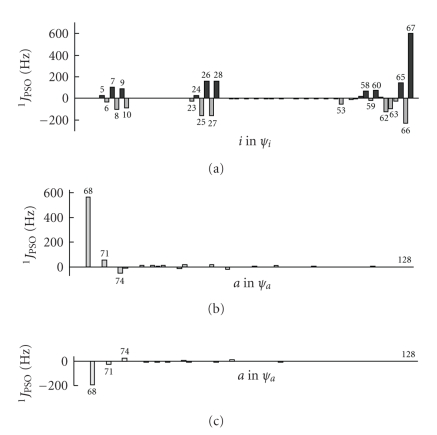
MO analysis of ^1^ *J*_PSO_ (Se, Se: **2a**): (a) contributions from each *ψ*_*i*_, (b) from each *ψ*_67_ → *ψ*_*a*_ transition, and (c) from each *ψ*_66_ → *ψ*_*a*_ transition.

The origin of ^1^ *J*(Se, Se: **2**) is elucidated by analyzing ^1^ *J*_PSO_ (Se, Se: **2a**) on the basis of the MO theory, since ^1^ *J*_PSO_ (Se, Se) contributes predominantly to ^1^ *J*_TL_ (Se, Se) irrespective of Y. Figure 7 depicts the contributions of ^1^ *J*_PSO_ (Se, Se: **2a**) separately from each *ψ*_*i*_ and each *ψ*_*i*_ → *ψ*_*a*_ transition. (a)–(c) in Figure 7 plot the contributions to ^1^ *J*_PSO_ (Se, Se: **2a**) from each *ψ*_*i*_ and each transition of the *ψ*_67_ → *ψ*_*a*_ and *ψ*_66_ → *ψ*_*a*_ types, respectively. In Figure 7(a), contributions around *ψ*_5_–*ψ*_10_, *ψ*_23_–*ψ*_28_, and *ψ*_53_–*ψ*_67_ originate mainly from atomic 2p(Se), 3p(Se), and 4p(Se) orbitals, respectively. Those caused by 2p(Se) and 3p(Se) are almost cancelled by summarizing over the corresponding orbitals. Therefore, 4p(Se) substantially contribute to ^1^ *J*_PSO_ (Se, Se: **2a**). Especially, *ψ*_67_ (HOMO) and *ψ*_66_ (HOMO-1) control ^1^ *J*_PSO_ (Se, Se: **2a**). *ψ*_*a*_ of *ψ*_68_ determines ^1^ *J*_PSO_ (Se, Se: **2a**), among a lot of *ψ*_*i*_ → *ψ*_*a*_ transitions in *ψ*_*i*_ of *ψ*_67_ and *ψ*_66_, as shown in Figures 7(b) and 7(c).

**Figure 8:**
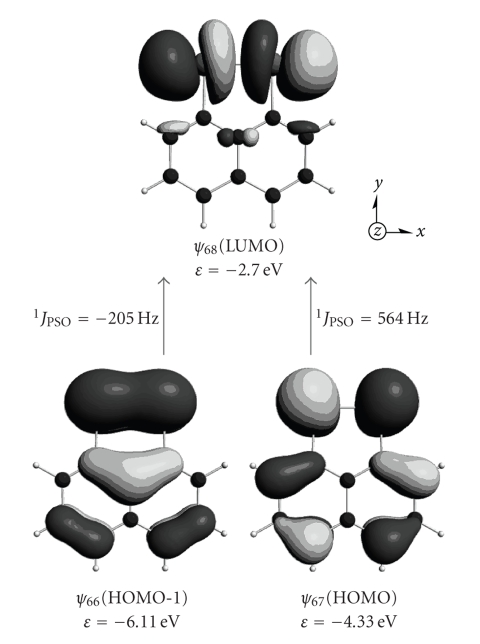
MO analysis of ^1^ *J*_PSO_ (Se, Se: **2a**): main contributions from the *ψ*_67_ (HOMO) → *ψ*_68_ (LUMO) and *ψ*_66_ → *ψ*_68_ transitions are depicted.

Figure 8 shows the *ψ*_67_ → *ψ*_68_ and *ψ*_66_ → *ψ*_68_ transitions in ^1^ *J*_PSO_ (Se, Se: **2a**). The large ^1^ *J*_PSO_ (Se, Se: **2a**) value arises from the mixing of *ψ*_68_ [LUMO: σ*(Se–Se)] into *ψ*_67_ [HOMO: π*(Se–Se)] and *ψ*_66_ [HOMO-1: *π*(Se–Se)] at the singlet state. The MO presentation in Figure 8 is essentially the same as the *ψ*_42_ → *ψ*_44_ and *ψ*_43_ → *ψ*_44_ transitions in ^1^ *J*_PSO_ (Se, Se: **1a**) at *ϕ* = 0° in Figure 4, although *ψ*_67_ (**2a**) and *ψ*_66_ (**2a**) contain the *π*(Nap) character. Large ^1^ *J*_PSO_ (Se, Se: **2**) and small ^1^ *J*_obsd_ (Se, Se: **1**) are well understood by the *ϕ* dependence in the calculated ^1^ *J*(Se, Se: **1a**) values.

## 4. Conclusion

Nuclear spin-spin coupling constants (*J*) provide highly important information around coupled nuclei, containing strongly bonded and weakly interacting states. The ^1^ *J*(Se, Se) values are analyzed as the first step to investigate the nature of the bonded and nonbonded interactions between the Se atoms through ^*n*^ *J*(Se, Se). QC calculations are necessary for the analysis and the interpretation of the *J* values with physical meanings. Calculated ^*n*^ *J*_TL_ are composed of the contributions from ^*n*^ *J*_DSO_, ^*n*^ *J*_PSO_, ^*n*^ *J*_SD_, and ^*n*^ *J*_FC_. The decomposition helps us to consider the mechanisms of the spin-spin couplings, which are closely related to the electronic structures of compounds. Main contributions are evaluated separately from each *ψ*_*i*_ and each *ψ*_*i*_ → *ψ*_*a*_ transition, where *ψ*_*i*_ and *ψ*_*a*_ are occupied and unoccupied MO's, respectively.

^1^ *J*(Se, Se) is calculated modeled by MeSeSeMe (**1a**), which shows the typical torsional angular dependence of *ϕ*(C_Me_SeSeC_Me_). The dependence explains well ^1^ *J*_obsd_ (Se, Se) of small values for RSeSeR′ (**1**) and large values for 4-Y-1,8-Se_2_C_10_H_5_ (**2**) which correspond to *symperiplanar* diselenides. ^1^ *J*_TL_ (Se, Se: **2**) are confirmed to be controlled by *Qn*(Se). ^1^ *J*_TL_ (Se, Se: **2**) are demonstrated to be smaller when *Qn*(Se) becomes larger, experimentally and theoretically. The PSO terms contribute predominantly to ^1^ *J*(Se, Se). The contributions are analyzed separately from each *ψ*_*i*_ and each *ψ*_*i*_ → *ψ*_*a*_ transition. The MO description of each transition enables us to recognize and visualize clearly the origin and the mechanisms of the indirect nuclear spin-spin couplings. Important properties of molecules, such as electronic structures, will be clarified by elucidating the mechanisms of the spin-spin couplings on the basis of the MO theory.

## Figures and Tables

**Table 1:** Observed ^1^ *J*_obsd_ (Se, Se) values of some selenium compounds.

Compound	^1^ *J*_obsd_(Se, Se) [Hz]	Comment
*t*BuSeSeMe	2.7	(a)
*n*BuSeSeMe	36.3	(a)
MeSeSePh	22	(a)
*o*-O_2_NC_6_H_4_SeSeCN	64	(b)
**2** (Y = Me)	379.4	This work
**2** (Y = Cl)	375.9	This work
**2** (Y = NO_2_)	330.8	This work

^(a)^ References [9, 16]. ^(b)^ References [9, 17].

**Table 2:** ^1^ *J*(Se, Se) values calculated for 1**a** ^(a), (b)^.

*ϕ*	*E*_rel_^(c)^	^1^ *J*_PSO_	^1^ *J*_SD_	^1^ *J*_FC_	^1^ *J*_SD+FC_	^1^ *J*_TL_
[°]	[kJ mol^−1^]	[Hz]	[Hz]	[Hz]	[Hz]	[Hz]
0.0	36.9	447.2	217.8	18.6	236.4	683.7
15.0	33.0	399.2	200.6	15.2	215.8	615.0
30.0	25.3	288.5	163.1	2.7	165.8	454.3
60.0	6.1	76.1	101.4	−43.3	58.1	134.2
75.0	0.9	20.0	87.8	−64.7	23.1	43.1
88.4	0.0	4.1	84.5	−76.7	7.8	11.9
90.0	0.0	4.2	84.6	−77.9	6.7	10.9
105.0	2.3	29.9	91.5	−77.4	14.1	44.0
120.0	7.4	94.7	109.3	−60.5	48.8	143.5
150.0	17.6	291.5	171.7	−8.2	163.5	455.0
165.0	21.5	370.6	201.1	9.0	210.1	580.7
180.0	22.8	400.7	213.4	14.3	227.7	628.4

^(a)^For the abbreviation, see text. ^(b)1^ *J*_DSO_ being less than 0.03 Hz. ^(c)^Relative to optimized value (−5267.7384 au) at *ϕ* = 88.38° in kJ mol^−1^.

**Table 3:** Contributions to the torsional angular dependence in ^1^ *J*_PSO_ (Se, Se: 1*a*) from *ψ*_*i*_^(a),(b)^.

*ϕ* [°]	0.0	15.0	30.0	60.0	75.0	88.4	90.0	105.0	120.0	150.0	165.0	180.0
*ψ*_1_–*ψ*_43_	447.2	399.2	288.5	76.1	19.9	4.1	4.3	29.9	94.7	291.4	370.5	400.7
*ψ*_39_–*ψ*_43_	449.9	403.1	294.9	84.4	35.2	20.4	20.6	45.9	109.1	302.2	380.3	410.4
*ψ*_39_	−121.2	−117.6	−108.9	−80.9	−63.0	−44.9	−42.6	−18.9	8.7	−146.9	−155.1	−157.7
*ψ*_40_	181.2	163.4	118.7	13.7	−28.6	−59.4	−62.8	−90.8	−114.3	65.3	85.4	93.0
*ψ*_41_	−43.3	−48.3	−60.8	−87.9	−94.3	−93.1	−92.4	−79.9	−54.0	28.7	62.4	75.0
*ψ*_42_	−359.7	−333.7	−266.1	−84.0	9.6	95.6	111.9	21.2	−71.9	−261.5	−328.1	−352.7
*ψ*_43_	792.9	739.4	612.0	323.7	211.5	122.2	106.5	214.3	340.7	616.6	715.7	752.8
*ψ*_42_ → *ψ*_44_^(c)^	−333.3	−307.1	−240.9	−69.4	15.6	93.3	116.5	33.2	−54.4	−235.6	−298.1	−321.0
*ψ*_43_ → *ψ*_44_^(c)^	747.2	695.7	574.7	312.4	206.8	125.9	103.2	202.0	320.7	581.5	673.8	708.6

^(a)^In Hz. ^(b)^For the abbreviation, see text. ^(c)^Contribution from the transition.

**Table 4:** ^1^ *J*(Se, Se) and *Qn*(Se) calculated on the full-optimized structure of 2^(a),(b),(c)^.

Compound	^1^ *J*_PSO_ [Hz]	^1^ *J*_SD_ [Hz]	^1^ *J*_FC_ [Hz]	^1^ *J*_SD+FC_ [Hz]	^1^ *J*_TL_ [Hz]	*Qn*(^1^Se)	*Qn*(^2^Se)	Symmetry
**2a**(Y = H)	362.2	195.2	−54.1	141.1	503.3	0.2367	0.2367	*C*_2*v*_
**2b**(Y = OMe)	394.3	207.5	−54.2	153.3	547.7	0.2256	0.2264	*C*_*s*_
**2c**(Y = Me)	363.6	195.1	−55.3	139.8	503.5	0.2334	0.2296	*C*_*s*_
**2d**(Y = Cl)	360.1	193.4	−53.1	140.3	500.4	0.2448	0.2443	*C*_*s*_
**2e**(Y = COOMe)	324.1	178.2	−55.7	122.5	446.6	0.2593	0.2556	*C*_1_
**2f**(Y = CN)	326.6	180.6	−52.6	128.0	454.6	0.2677	0.2564	*C*_*s*_
**2g**(Y = NO_2_)	299.7	167.6	−53.9	113.7	413.4	0.2824	0.2576	*C*_1_
**2a**(Y = H)^(d)^	390.7	206.4	2.6	209.0	599.7	0.2367	0.2367	*C*_2*v*_

^(a)^For the abbreviation, see text. ^(b)1^ *J*_DSO_ being less than 0.03 Hz. ^(c)1^Se and ^2^Se being attached to ^1^C and ^8^C in 4-Y-1,8-Se_2_C_10_H_5_ (**2**), respectively. ^(d)^On the basis of scalar ZORA.
